# An Aerial Voyage

**Published:** 1871-10

**Authors:** 


### AN AERIAL VOYAGE.

So little advance has been made in the science of aëronautics during the past century, and so rare is the occurrence of a successful balloon ascension, that when such a spectacle is offered the inhabitants of a city, it is welcomed with more than ordinary interest. We have had an unusual opportunity for discovering that much curiosity is experienced, by all classes of community, to know what the particular sensations are that accompany an aërial voyager,—a universal desire to know what he sees, feels, and thinks, while scudding through mid air in so frail a bark as a balloon. This article will, therefore, be an attempt at describing an aërial voyage, being conscious the while, that any sketch of ours must fall far short of the sublime grandeur of the reality. It is one of those experiences that cannot be described—the impressions that quicken our senses are entirely new, leaving us at a loss for familiar phrases of illustration.

The desire to take an aërial trip was not new to us,—for years we had thought of undertaking the journey, when an opportunity offered so to do, with either of the scientific Aëronauts, Profs. King or Wise. When, therefore, it was announced that Prof. SAMUEL A. KING was to make a balloon ascension from our city, on the second day of September last, we at once secured passage in his beautiful balloon, the "Aurora."

This balloon, when fully inflated, stands fifty feet high, and is thirty feet in diameter, holding 20,000 feet of gas. When filled with carbureted hydrogen or common burning gas, which is only one-half lighter than air, it is capable of raising, independent of its own weight, (which is 325 lbs.), 350 pounds, to the height of one and a half miles. If inflated with hydrogen gas, which is 14.4 lighter than air, it will carry a considerably heavier load to a greater height.

The day selected for the ascension was a beautiful one,—not a cloud was to be seen, scarcely a breath of air stirring, and the temperature comfortable at eighty degrees. Our ballast consisted of three small bags of sand, weighing about seventy-five pounds, a package of circulars of thirty pounds weight, a small pail of water, two overcoats, a bottle of champagne, and a luncheon. The balloon was anchored to the ground by means of several heavy sand-bags, attached to the netting. These were removed after taking our position in the car, while a half-dozen men held us down by means of a rope attached to the rigging. At 3:05 P. M., everything being in readiness, and the spectators directed to stand away from the balloon, the Prof. detached the anchor-rope, when, like a thing of life, it rose majestically into the air, while the people expressed their delight and admiration, by loud and repeated huzzas.

At the start, we occupied a safe position in the bottom of the car, for prudential reasons, as experience had taught us that we could not look from a high building or cliff, without an almost ungovernable desire to leap off; neither can we climb a high ladder without being more or less dizzy; while swinging is particularly distasteful to us. Therefore, as we cautiously and with some timidity, looked over the side of the car, we were surprised to find that none of these uncomfortable sensations manifested themselves. We were astonished to see our friends, with the earth, sink rapidly away from us, their voices growing indistinct, their stature fast becoming lilliputian, while *we* seemed to be *in situ*, no motion being perceptible in the balloon.—Strange to say, at this great height, no dizziness or fear manifested itself, and it was difficult to believe otherwise than that we were upon some firm and secure lookout, watching the wonderful disappearance, into unfathonable depths below, of creatures and objects terrestial. When our barometer had indicated an altitude of one mile, we found a current of air that carried us to the south-west. This fact was perceptible only as we discovered the earth, and the circulars which we threw from the balloon, moving in an opposite direction; no breeze, not a rustle of a bit of paper, or the motion of anything, indicated the presence of such a current,—everything about us was serene. We could now hear the voices of children, the crowing of cocks, steam whistles, and occasionally the shrill voice of a woman calling the attention of some one to the balloon. All other sounds seemed to be shut out from us. Men, when directly under us, looked as though trying to extricate themselves from beneath the ground, their hats and shoulders only being visible. Cows looked like monstrous insects crawling over the surface of the earth, their horns and broad ears corresponding to, and resembling closely, the antenniæ. Sheep were skipping about like grass-hoppers, and horses and wagons looked like toys, drawn by some mysterious power, as neither legs nor wheels were visible. Fields of grass were smooth and velvety, corn-stalks resembled grass, and trees had the appearance of low under-brush; houses and barns, when seen at an angle, resembled the toy-houses of children, but when looked upon perpendicularly, looked like small roofs laying upon the surface of the ground.

We continued sailing along at this height and direction, until we were two-thirds the distance to Wellsburg, a village six miles south of the city, when we discovered that the direction of the current was such as to carry us out of reach of a railroad. Prof. KING, therefore, determined to change the course of the balloon, and astonished us with the information that he intended sailing back to the city again; for, said he, "If I can get far enough to the north-west, I can drop into our present current again and return immediately over the city, so that the inhabitants can have a fine view of us."

"Sail over the city *again!*" we exclaimed.

"Certainly," he replied.

"But how?" we inquired.

"O, bless you, I'll show you—all easy enough! You see that we are now moving in a south-western current; I so determine by watching two objective points on earth, and observing our deviation from a line between them. You see that bare spot on top the mountain yonder? The compass shows that it is directly south of us. Now look immediately under us,—see that farm-house just on a line with the edge of the car,—now, don't lose sight of those two points, else we'll lose our reckoning also. It is now 3:40,—we have been thirty-five minutes coming about four miles; slow traveling, you think; I *have* traveled eighty miles an hour in this same balloon, but that was in a gale of wind, the riding of which was more rapid and terrific, than safe or comfortable. See where your farm-house *now* is!"

"Where? *I* can't see it at all!"

"Why, there, to the north-east of us, while the hill is to the south east;—we're going nearly west now."

"But I saw the hill over *my* end of the car, while now it's over *yours!*".

"O, that is no matter; the balloon has revolved once since you looked before."

"But I didn't *see* it turn;—say, isn't this just a little confusing? how in fury did Wellsburg and Elmira swap places so quickly?"

"The revolutions of the balloon are always confusing to one's idea of direction,—you see it revolves so easily and smoothly that you don't discover it unless you are watching one object constantly."

"But you said we should return to Elmira."

"So we will; we must first throw over sand, so as to ascend and strike the reverse current, which we will surely find some-where above us. Observe the sand falling—looks like a stream of black ink, and by the time it reaches earth, will be so thoroughly distributed as to do injury to no one.

"Now throw over a lot of those circulars,—see, they fall like lead,—all right, we're going up! Doubtless, if our friends are watching us, they can see that we're rising. *Now* take a look out,—what do you think of the prospect?"

We looked, and behold, all things had changed! The view that met our gaze was indescribably grand and beautiful. The horizon, completely encircling us, looked as though we were surrounded by an immense snow-bank, miles upon miles in extent; occasionally a cloud could be seen, white as the purest snow, with its edges gaily trimmed with green and gold. Looking aloft, the balloon seemed aglow with all the brilliant tints of the rain-bow, while the sky had lost its azure hue, and presented a vivid brightness too dazzling to look upon. A feeling of indescribable loneliness took possession of us when, looking below, we found that objects upon earth had vanished into undistinguishable atoms. The highest mountains and lowest dales were transformed into one vast plane. The Chemung River, Seely and Newtown Creeks lay sparkling in the sunlight at our feet, entwined among golden fields of grain, beautifully and variously tinted fields of green, with here and there the darker hues of a deeply shaded hillside; the whole interspersed with our public thoroughfares, with now and then a lake, island, woodland, and village; all brilliantly lighted by the direct rays of the sun, presented an appearance, from our aërial observatory, as though looking upon an exquisitely-wrought picture, from a master-hand; or, upon some immense park artistically planned and beautified by a marvelously skilled landscape-gardener. Away to the north could be seen Seneca, Keuka, Canandaigua and Cayuga Lakes, reflecting the sunlight back to us in a peculiar, silvery whiteness, as though from the surfaces of highly polished mirrors. The margins of the lakes could readily be discerned, enabling us to trace them to their extreme northerly limit, more than sixty miles away. To the south of us was seen the village of Waverly, with its white houses peeping out from the green foliage with which they seemed to be surrounded. To the left of it lay the little village o Athens, with the sparkling Susquehanna River beyond. To the south-west, and nearer to us, was seen Chemung, and nearly under us, Wellsburg, at which point our river appeared as though completely covered with little,twinkling stars. Away to the north-west lay Bath, Corning and Painted Post; to the north, Horseheads, Millport and Watkins; to the north-east, Breesport, Van Ettenville, and other villages which we did not recognize, but all of which were plainly discernable to the naked eye, presenting a picture long to be remembered, and never to be forgotten.

We note the change in the temperature, and looking at the thermometer find that it has fallen 30°. The air has become rarer, producing an uncomfortable sensation about our ears. Our voices sound as though uttered in some deserted hall, while our laugh is rendered musical and merry from the balloon above. Sounds from earth have entirely disappeared, and throwing over a bundle of circulars reveals, the fact that we are still rising, and drifting toward the city from whence we started. We look at the barometer; it indicates an altitude of 8,000 feet—more than one mile and a half! How quiet is everything about us! We imagined, when a mile nearer the earth, that we were then in the midst of perfect stillness; but that was wild confusion compared with the absence of all sound, which we experienced at this great height. The stillness becomes startling—painful in fact, so that we unconsciously commence the humming of a song to assure ourself that we have not lost the power of creating sounds. We find, as our song progresses, that it is that peculiarly appropriate one, in keeping with the eternal fitness of things:—

"Up in a balloon, boys, up in a balloon;
All among the little stars, sailing 'round the moon."

From our note book we learn that we had gained our greatest altitude at 4:35, and were about a mile south-west of the city, on our return trip. At this point we concluded to test the champagne, thoughtfully provided us by our friend, Major Caldwell. Champagne that is moderately effervescing upon earth, becomes furious when uncorked in the sky. We think we could easily have supplied a company of soldiers with well filled glasses, from one bottle of champagne;—the drink, doubtless, would have been rather *thin*, but possessing the single merit of being sufficiently sparkling.

We continued at this altitude, passing over East Hill, immediately to the rear of Rev. Thos. K. Beecher's residence, at 4:50. At 5:20 we stood abreast of Horseheads, and immediately south of Breesport. The country presented a beautiful appearance in this direction; the bright wheat-fields, alternating with the dark dots of woodland, a majority of them being square, reminded one of an immense chess-board. We now found that our gas had so expanded as to overflow from the neck of the balloon, rendering our breathing at times, somewhat difficult. Considerable gas being so discharged, we soon fell into the lower current again, when our direction was reversed, carrying us to the south-west. We had now fallen to within speaking distance of the people below us, and carried on a conversation at every farm-house over which we passed. Several persons ran after us, along the road, asking us all manner of questions. As they ran they were reinforced by others who joined in the chase. Their motions were very comical, looking to us as though their arms moved faster, and had more to do in propelling their bodies, than their legs.

At an altitude of about 3,000 feet we find a shrill, musical sort of an echo from the earth, which is marvelous for its distinctness. In uttering a sentence of about a dozen words, in rather a loud tone of voice, by the time the last word of the sentence has left your lips, you hear the first one from the echo, each alternate one being distinctly given, to the last. This was a source of great amusement to us, and we continued our conversation, laughter, singing, and whistling, until we had descended so far as to lose it entirely. The echo of our laughter was so rollickingly jolly, that one was forced to laugh in response to it, whether he would or no.

We soon fell into the shade of the mountain, which so condensed our gas as to cause the balloon to descend very rapidly. Having disposed of all our sand, we found it necessary to throw over our champagne bottle, glasses, luncheon, and water, in order to lighten the balloon sufficiently to enable us to clear the treetops. This aided us for a few minutes, but we soon descended again, when our "drag-rope," 150 feet long, was thrown over so as to rest upon the trees. This again lightened us, notwithstanding it was dragging over the tops of the trees for more than fifty feet behind us. Finally, our basket struck the top of a large spruce tree, from which we bounded like an india-rubber ball, immediately descending again to strike another; bounding along for a few minutes in this manner was rare sport, but became alarming when we saw a large, dead hemlock tree, with sharp, bristling branches, just ahead of us. Over went our overcoats in a moment, when up we went again, clearing the dreaded obstacle beautifully. We saw a little hamlet just ahead of us, and are informed by one of our followers that it is Hammond's Corners, just seven miles from Elmira. We see a good road leading down through the valley, and an abundance of help having arrived, we determine to descend as soon as we can find a clear space, sufficiently large, to enable us so to do. Presently, we see a field over which we are likely to pass, and halloo to several men near, to run to it and be prepared to grasp our drag-rope. They comprehend the situation of affairs at once, and are soon upon the ground, ready to receive us. As our rope falls from the trees to the ground, into the open space, it is grasped by stout hands, when the further progress of the aërial ship is brought to a stand-still. Soon, a large force is collected about us, we are drawn to the ground, and alight from our car at 6:12, without an accident of any kind to mar the pleasure of the most enjoyable voyage of our life.

The good citizens of Hammond's Corner's assisted us to pack our balloon, furnished us with an excellent supper, and supplied us with a conveyance to bring ourselves and balloon to the city, where we arrived at eleven o'clock, welcomed by our friends, who gave glowing accounts of the beautiful ascension as it appeared to them from earth, all agreein that it was by far, the most successful one ever made from our city.

And so ended our first aërial flight—an experience that never can be effaced from our memory, and we hope, whenwe repeat it, that it may be under as good management, and favorable circumstances, as the present one; whenwe expect to be able to give our readers the result of certain scientific observations, which it is our desire to make.

---

THE tobacco factories of New York and Brooklyn employ 20,000 children.

